# Alkannin Protects Against UVB-Induced Skin Photoaging by Targeting Keap1 to Activate the Nrf2/HO-1 Pathway

**DOI:** 10.3390/molecules31081278

**Published:** 2026-04-13

**Authors:** Qilong Wu, Feiping Tao, Nan Zhang, Yong Li, Shuwei Li

**Affiliations:** 1 State Key Laboratory Incubation Base for Conservation and Utilization of Bio-Resource in Tarim Basin Xinjiang Production & Construction Corps, College of Life Sciences and Technology, Tarim University, Alar 843300, China; qlw_12936@163.com (Q.W.); a2090314329@126.com (F.T.); zhangnan_brave@163.com (N.Z.); ly_717192@163.com (Y.L.); 2 College of Basic Medicine, Shanxi University of Chinese Medicine, Fenyang 032200, China

**Keywords:** Alkannin, photoaging, Keap1, Nrf2/HO-1 pathway, HaCat cell, ultravioletB

## Abstract

Ultraviolet B (UVB), as a major component of solar radiation, is a key factor in inducing skin photoaging. The epidermis serves as the primary defensive barrier of the skin and absorbs the majority of UVB. This study aims to elucidate the protective effect of Alk against UVB-induced photoaging and further uncover its underlying molecular mechanisms. In vitro, Alk-pretreated HaCaT cells were exposed to UVB. Cell viability, ROS, senescence, antioxidant enzymes, and protein expression were analyzed. Mechanisms were examined using CETSA, DARTS, Co-IP, and NRF2 knockout. In vivo, Alk hydrogel was tested in UVB-exposed BALB/c mice, with protection assessed via histology and immunohistochemistry. In vitro, Alk directly binds to Keap1, disrupts Keap1–Nrf2 interaction, promotes nuclear translocation of Nrf2, and upregulates the expression of its downstream target HO-1. Consequently, intracellular ROS generation is reduced, cellular senescence is alleviated, and the expression of inflammatory factors (TNF-α, COX-2) and MMP-9 is suppressed. In vivo, topical application of the Alk hydrogel prevented UVB-induced skin thickening and collagen degradation. Alk exerts a preventive effect on UVB-induced photoaging in HaCaT cells and skin, providing strong support for developing Alk as a potential plant-derived active ingredient for preventing skin photoaging.

## 1. Introduction

The skin is directly exposed to ultraviolet radiation (UVR) from sunlight, providing the outermost layer of protection for the human body. UVR consists of three subtypes: long-wave ultraviolet A (UVA, 320–400 nm), mid-wave ultraviolet B (UVB, 280–320 nm), and short-wave ultraviolet C (UVC, 200–280 nm). UVC is completely blocked by the ozone layer, while UVA and UVB can penetrate the atmosphere and directly impact the human body [1]. Although UVB constitutes only approximately 5% of total UVR, it is responsible for more oxidative damage than UVA [2]. Primarily absorbed by the epidermis, UVB causes skin redness, laxity, promotes epidermal thickening, and leads to collagen fiber degradation. Overexposure to UVB irradiation leads to a significant increase in intracellular reactive oxygen species (ROS), thereby inducing oxidative stress in cutaneous tissue [3]. The continuous accumulation of ROS can damage lipids and proteins, impair antioxidant enzyme activity, and ultimately result in oxidative stress [4]. This subsequently induces the upregulation of inflammatory cytokines and matrix metalloproteinases (MMPs) [5], culminating in collagen breakdown. The nuclear factor erythroid 2-related factor 2 (Nrf2) signaling pathway is a classical oxidative stress response pathway. Cells constantly synthesize Nrf2, which is bound in the cytoplasm by Kelch-like ECH-associated protein 1 (Keap1), targeting it for ubiquitin-mediated degradation [6]. It has been reported that certain small molecules can bind to Keap1, disrupting the Keap1–Nrf2 complex and thereby promoting Nrf2 nuclear translocation [7,8]. Once in the nucleus, Nrf2 upregulates the expression of downstream antioxidant enzymes such as heme oxygenase-1 (HO-1). Elevated HO-1 activity enhances the cellular antioxidant defense system, effectively scavenging excess ROS and mitigating oxidative damage.

*Lithospermum erythrorhizon*, known as “Zicao” in traditional Chinese medicine, has a documented history of over two thousand years [9]. It has been widely used to treat burns, scalds, psoriasis, and other conditions. Alkannin (Alk) is extracted from the dried roots of Xinjiang Arnebia or Inner Mongolia Arnebia. Alkannin and its enantiomer shikonin are isomers belonging to the naphthoquinone class of compounds [10]. Alkannin is a key pharmacologically active component of *Lithospermum erythrorhizon* and has been shown to possess anti-inflammatory, antioxidant, and wound-healing activities [11]. Shikonin has been found to inhibit oxLDL-mediated ROS production by activating the PI3K/Akt/Nrf2 pathway [12]. Furthermore, studies indicate that Alkannin can protect keratinocytes from UVB radiation-induced apoptosis by inducing the expression of HSP70 [13]. However, how Alkannin alleviates skin photoaging remains incompletely understood.

Therefore, this study utilized UVB-induced photoaging models in both HaCaT cells and BALB/c mice to systematically evaluate the protective efficacy of Alk and investigate the underlying mechanisms of its anti-photoaging effects.

## 2. Results

### 2.1. Alk Inhibits UVB-Induced Damage and Senescence in HaCaT Cells

**Figure 1:**
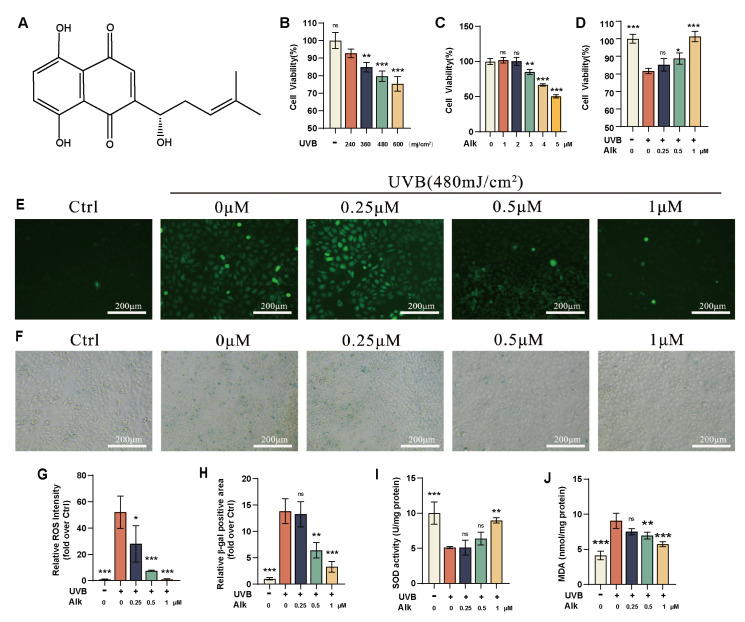
Alk inhibits UVB-induced damage and senescence in HaCaT cells. (**A**) Molecular structural formula of Alk. (**B**,**C**) The effects of different irradiation doses and varying concentrations of Alk on the viability of HaCaT cells. (**D**) The impact on HaCaT cell viability in cells pre-treated with Alk and then exposed to UVB. (**E**,**F**) Intracellular ROS and SA-β-gal staining; ROS levels were measured 1 h after UVB irradiation, and SA-β-gal staining was performed 24 h after UVB irradiation; scale bar = 200 μm. (**G**) Relative fluorescence intensity of ROS was analyzed using ImageJ. (**H**) Relative positive area of SA-β-gal staining was quantified with ImageJ. (**I**) Cellular SOD activity was measured 24 h after UVB irradiation. (**J**) Cellular MDA content was measured 24 h after UVB irradiation. Data are presented as mean ± SD (*n* = 3 independent experiments, with 3 technical replicates per experiment). * *p* < 0.05, ** *p* < 0.01, *** *p* < 0.001.

We first determined the suitable UVB irradiation dose for establishing the photoaging model. Statistical data showed that cell viability significantly decreased when the irradiation intensity reached 480 mJ/cm^2^. (Figure 1B), Therefore, we selected 480 mJ/cm^2^ as the irradiation intensity for model establishment. We evaluated the cytotoxicity of Alk on HaCaT cells and observed that cell viability began to decrease significantly when the concentration of Alk reached 3 μM (Figure 1C). Consequently, a non-cytotoxic concentration of 1 μM was chosen for further studies. The pre-protective effect of Alk (0.25, 0.5, and 1 μM) against irradiation in cells alleviated irradiation-induced damage in a dose-dependent manner, as reflected by increased cell viability (Figure 1D). Given the pivotal role of ROS in mediating oxidative stress during photoaging, we performed the following: After treatment with a gradient of Alk concentrations, the expression of ROS in irradiation-induced cells was measured using fluorescence staining. The results showed that compared with the control group, UVB treatment activated intracellular ROS, resulting in a marked elevation in fluorescence intensity, while Alk treatment reversed this trend, leading to a significant increase (Figure 1E,G), indicating the antioxidant capacity of Alk. To evaluate the effect of Alk on irradiation-induced senescence in cells, we used SA-β-Gal staining to assess the cellular senescence status. The staining results showed the number of senescence-positive cells increased significantly after UVB irradiation, whereas treatment with Alk reduced the generation of positive cells (Figure 1F,H), suggesting an anti-senescence role of Alk. In addition, measurements of SOD activity and MDA content showed that UVB irradiation decreased SOD activity and increased MDA levels; both effects were reversed by Alk treatment (Figure 1I,J).This set of findings demonstrates that Alk effectively suppresses oxidative damage and cellular senescence triggered by UVB in HaCaT cells.

### 2.2. Alk Alleviates UVB-Induced Photoaging by Activating the Nrf2-HO-1 Pathway

**Figure 2:**
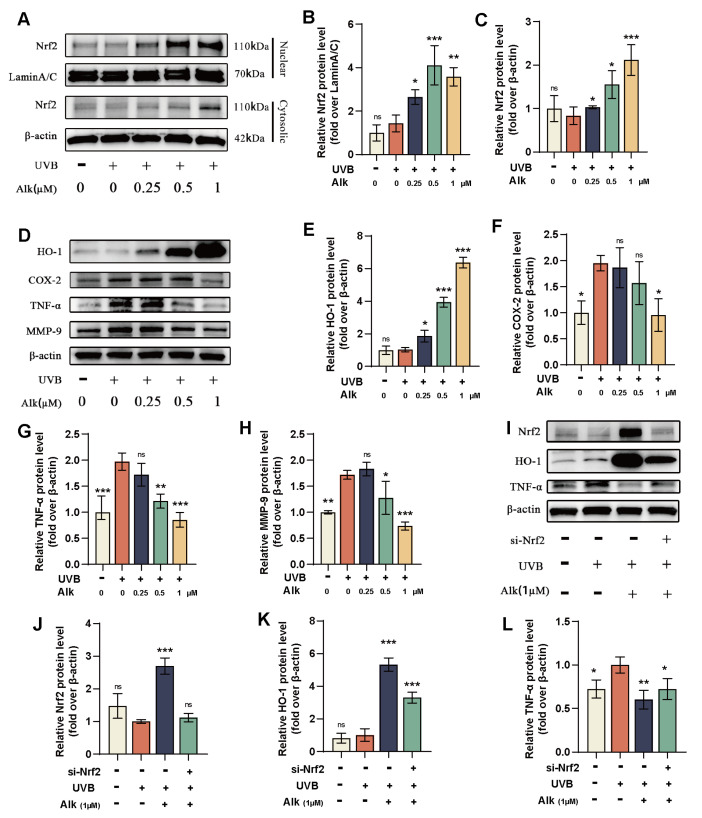
Alk alleviates UVB-induced photoaging by activating the Nrf2-HO-1 pathway. (**A**) Western blot analysis of Nrf2 expression in the nucleus and cytoplasm after treatment with Alk and UVB for 1.5 h. (**B**,**C**) Relative expression levels of Nrf2 in the nucleus and cytoplasm. (**D**) Western blot analysis of HO-1, COX-2, TNF-α, and MMP-9 protein levels. (**E**–**H**) Relative expression levels of HO-1, COX-2, TNF-α, and MMP-9. (**I**) Western blot analysis of Nrf2, HO-1, and TNF-α protein levels. (**J**–**L**) Relative expression levels of Nrf2, HO-1, and TNF-α. Data are presented as mean ± SD (*n* = 3 independent experiments, with 3 technical replicates per experiment). * *p* < 0.05, ** *p* < 0.01, *** *p* < 0.001.

Our above findings show that Alk reduces ROS production and enhances cellular antioxidant capacity, suggesting its potential to activate antioxidant stress response pathways. Given that nuclear translocation of Nrf2 is a critical step in upregulating cellular antioxidant defenses, To investigate whether Alk exerts its protective effect through Nrf2, we extracted proteins from the nucleus and cytoplasm separately and performed analysis by WB. As shown in (Figure 2A–C), Alk promotes the nuclear translocation of Nrf2 in HaCaT cells. In addition, the expression level of HO-1, a downstream protein of Nrf2, also exhibited a dose-dependent upregulation with increasing concentrations of Alk (Figure 2D,E). UVB damage can induce cells to secrete inflammatory factors and matrix metalloproteinases. To evaluate whether Alk can inhibit this condition, we examined the following three proteins: COX-2 and TNF-α are central mediators of inflammatory responses. A pronounced upregulation of COX-2 and TNF-α was observed in HaCaT cells following UVB irradiation. However, treatment with Alk (1 μM) effectively mitigated these increases (Figure 2D,F,G). MMP-9, an enzyme responsible for collagen degradation and cellular senescence, was also significantly elevated following UVB exposure. Treatment with medium and high concentrations of Alk effectively suppressed this UVB-induced upregulation of MMP-9 (Figure 2D,H). These results collectively demonstrate the protective role of Alk in UVB-irradiated HaCaT cells. To determine the dependency of Alk’s effects on Nrf2, we utilized Nrf2-specific small interfering RNA (siRNA) to knock down its expression in HaCaT cells. Transfection with si-Nrf2 successfully reduced Nrf2 protein levels even after Alk treatment (Figure 2I,J). As expected, Nrf2 knockdown suppressed the activation of its downstream target HO-1. Interestingly, in si-Nrf2 transfected cells, Alk treatment still induced a modest but observable increase in HO-1 expression compared to the corresponding control (Figure 2I,K). This residual upregulation suggests that Alk might partially activate HO-1 through alternative pathways. Notably, shikonin, an isomer of Alk, has been reported to activate the HIF-1α signaling pathway, which could represent one such parallel mechanism. Furthermore, knocking out Nrf2 reversed the anti-inflammatory activity of Alk, as evidenced by the fact that silencing Nrf2 abolished the Alk-induced decrease in TNF-α protein levels in HaCaT cells (Figure 2I,L). Collectively, these data suggest that Alk exerts its protective effect against UVB-induced photoaging by activating the Nrf2/HO-1 pathway.

### 2.3. Alk Promotes Nrf2 Nuclear Translocation by Directly Targeting Keap1

**Figure 3:**
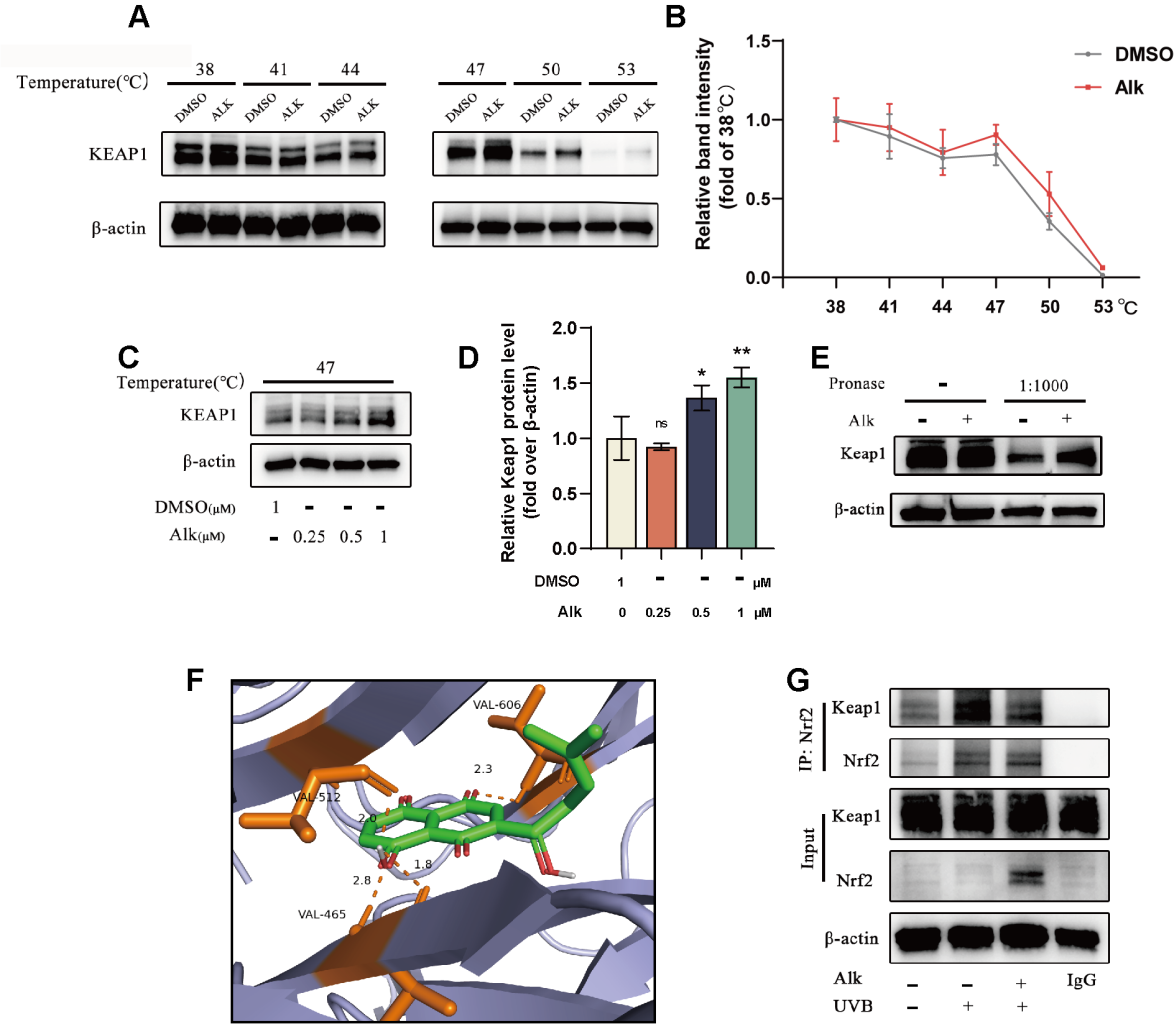
Alk promotes Nrf2 nuclear translocation by directly targeting Keap1. (**A**) Analysis of the interaction between Alk and Keap1 in HaCaT cells using the CETSA. (**B**) The relative Keap1 protein levels at various temperatures compared to the level at 38 °C. (**C**,**D**) Keap1 protein stability at 47 °C under different concentrations of Alk treatment. (**E**) DARTS assay demonstrating the interaction between Alk and the Keap1 protein in HaCaT cells. (**F**) Molecular docking results predicting the binding mode between Alk and the Keap1 protein. (**G**) Co-IP assay for Alk-Keap1-Nrf2 interaction. Data are presented as mean ± SD (*n* = 3 independent experiments, with 3 technical replicates per experiment). * *p* < 0.05, ** *p* < 0.01.

Nrf2 undergoes ubiquitination-mediated degradation upon binding to Keap1, which prevents its nuclear translocation and subsequent transcriptional activation. To investigate whether Alk exerts its effect by binding to Keap1 and disrupting the Keap1-Nrf2 interaction, we performed molecular docking simulations. The results predicted that Alk potentially forms interactions with residues VAL-465, VAL-512, and VAL-606 of the Keap1 protein (Figure 3F). The CETSA and isothermal dose–response experiments indicated that Alk dose-dependently enhanced the thermal stability of Keap1 (Figure 3A–D). Furthermore, DARTS data demonstrated that Alk treatment suppressed the degradation of Keap1 protein by Pronase E (Figure 3E). Co-IP analysis confirmed that Alk treatment inhibited the formation of the Keap1-Nrf2 complex in HaCaT cells (Figure 3G). Based on these collective findings, we propose that Alk directly binds to Keap1, disrupts the Keap1-Nrf2 interaction, and thereby leads to the buildup of Nrf2 and subsequent nuclear translocation.

### 2.4. Alk Protects Mice from UVB-Induced Skin Photoaging

**Figure 4:**
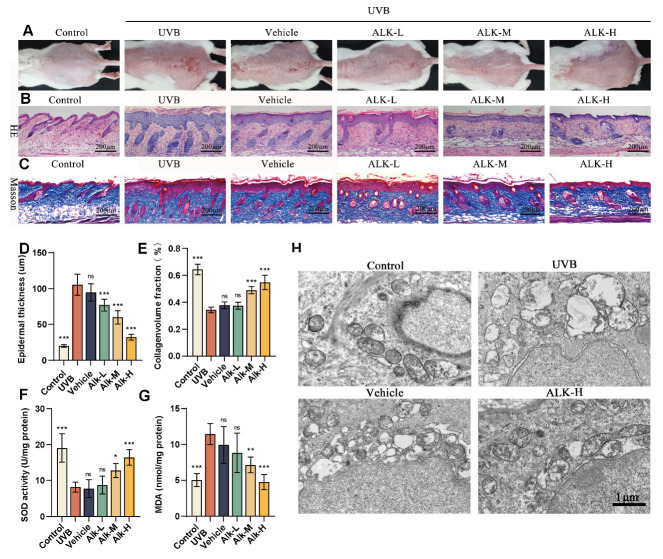
Alk protects mice from UVB-induced Skin photoaging. (**A**) Gross observation of BALB/c mice. (**B**,**C**) Histological morphology observed by H&E and Masson staining; scale bar = 200 μm. (**D**,**E**) Epidermal thickness and collagen volume fraction quantification. (**F**,**G**) SOD and MDA assays in skin tissues. (**H**) Epidermal ultrastructure in BALB/c mice (TEM). Scale bar = 1 μm. Data are presented as mean ± SD (*n* = 5 mice per group). * *p* < 0.05, ** *p* < 0.01, *** *p* < 0.001 versus the UVB group.

We determined an irradiation-induced photoaging model in BALB/c mice to evaluate the preventive efficacy of Alk. After six days of UVB exposure, the skin of mice in the UVB, Vehicle, and Alk-L groups exhibited significant redness, scaling, edema, and erosion. In contrast, these symptoms were markedly alleviated in the Alk-M and Alk-H groups (Figure 4A). As evidenced by H&E staining, Relative to the UVB group, the Vehicle group showed no alleviation effect, while the Alk hydrogel groups exhibited a dose-dependent reduction in epidermal thickness (Figure 4B,D). Furthermore, Masson’s trichrome staining demonstrated that the Alk hydrogel dose-dependently mitigated collagen degradation (Figure 4C,E). Measurement of SOD activity and MDA content in skin tissues showed that both SOD activity was decreased and MDA content was increased in the UVB and Vehicle groups. Alk treatment effectively reversed these changes in a dose-dependent manner (Figure 4F,G). Transmission electron microscopy show reduced mitochondrial cristae and efflux of cellular contents in the UVB and Vehicle groups. These ultrastructural abnormalities were notably reversed in the Alk-H group (Figure 4H). These results collectively indicate that the topical Alk hydrogel exerts a protective effect against irradiation-induced skin photoaging in mice.

### 2.5. Alk Alleviates UVB-Induced Photoaging by Inhibiting TNF-α and MMPs

**Figure 5:**
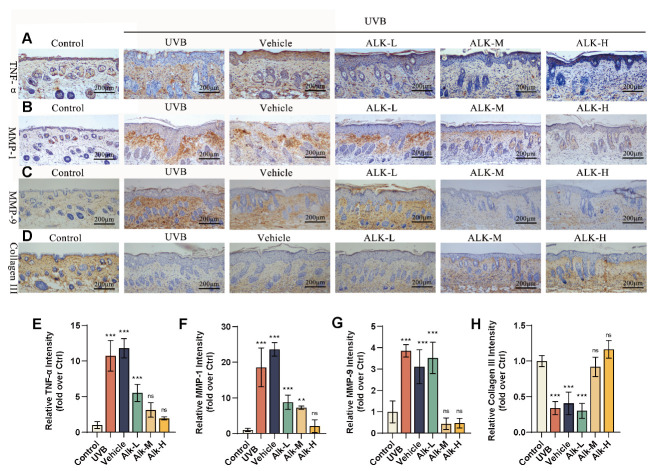
Alk alleviates UVB-induced photoaging by inhibiting TNF-α and MMPs. (**A**–**D**) Representative immunohistochemical staining images showing the abundance of TNF-α, (**B**) MMP-1, MMP-9, and Collagen III in mouse dorsal skin sections. Scale bar = 200 μm. (**E**–**H**) Staining intensity quantification for TNF-α, MMP-1, MMP-9, and Collagen III. Data are presented as mean ± SD (*n* = 5 mice per group). ** *p* < 0.01, *** *p* < 0.001.

To further demonstrate that the Alk hydrogel can prevent skin photoaging in mice, we analyzed the abundance of TNF-α, MMP-1, MMP-9, and Collagen III in mouse skin using immunohistochemistry. TNF-α is a pivotal cytokine that initiates inflammatory responses. The results showed a significant upregulation of TNF-α expression following UVB irradiation. Treatment with the Alk hydrogel attenuated the elevated expression in a concentration-dependent manner (Figure 5A,E). Matrix metalloproteinases (MMPs) break down key structural components including collagen and elastin, thereby contributing to the development of wrinkles and reduced skin firmness. Relative to controls, both the UVB and Vehicle groups exhibited a significant upregulation in the expression of MMP-1 and MMP-9. In contrast, the Alk hydrogel groups exhibited a dose-dependent reduction in the protein expression of both MMP-1 and MMP-9, thereby mitigating damage to the dermal collagen fibers (Figure 5B,C,F,G). Collagen III is an important reparative collagen in the skin. A reduction in its expression was observed in the UVB, Vehicle, and Alk-L groups (Figure 5D,H), indicating impaired dermal structure and synthetic function. These results collectively demonstrate that the Alk hydrogel effectively alleviates skin photoaging by suppressing the expression of TNF-α and MMP proteins.

### 2.6. Transcriptomic Analysis

**Figure 6:**
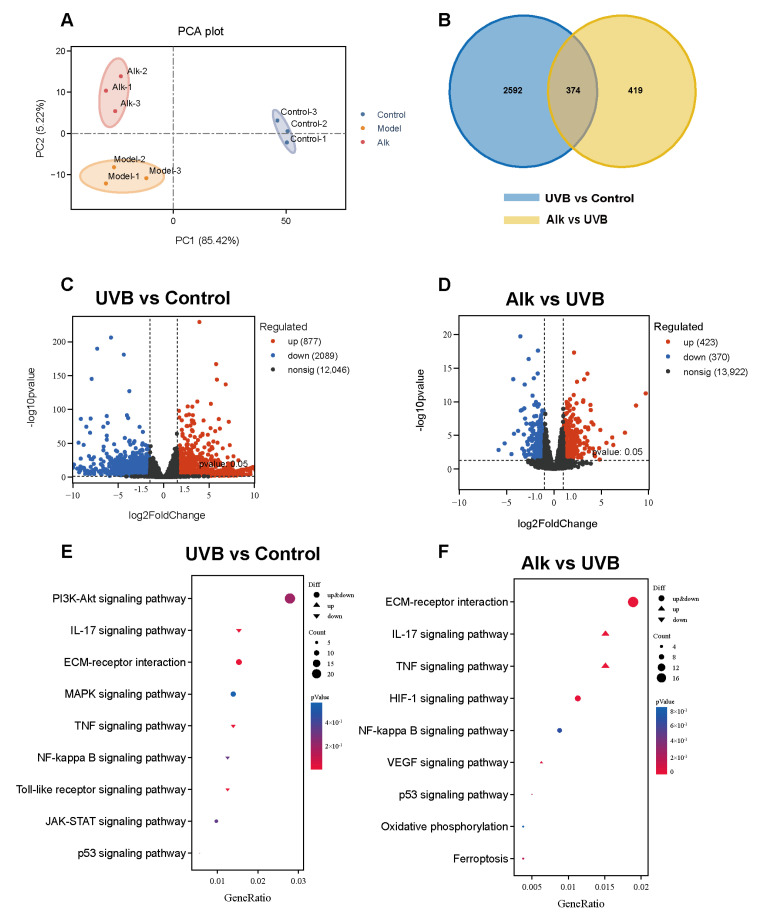
Transcriptomic Analysis. (**A**) Transcriptomic PCA depicting the separation among Control, UVB, and Alk-treated groups. (**B**) Overlap analysis of differentially expressed genes via Venn diagram for the indicated pairwise comparisons. (**C**,**D**) Volcano plots representing DEGs in the UVB vs. Control and Alk vs. UVB groups. (**E**,**F**) KEGG pathway analysis of enriched terms for the UVB-Control and Alk-UVB comparisons.

Transcriptomic profiling was performed on skin tissues collected from the Control, UVB, and Alk groups via RNA sequencing. PCA clearly separated the three groups based on their global gene expression patterns (Figure 6A). Venn diagram analysis revealed 374 significantly differentially expressed genes between the UVB + Control group and the Alk + UVB group (Figure 6B). According to the volcano plots, the UVB group had 877 upregulated and 2089 downregulated genes relative to the Control group (Figure 6C). In contrast, the Alk group showed a profile of 423 upregulated and 370 downregulated genes relative to the UVB group (Figure 6D). KEGG pathway enrichment analysis demonstrated that the IL-17 signaling pathway and the TNF-α signaling pathway were significantly enriched among the upregulated genes in the UVB versus Control comparison. Conversely, the Alk group showed suppressed activity in these pathways relative to the UVB group (Figure 6E,F). This transcriptomic evidence further supports the conclusion that Alk confers protection against UVB-induced skin photoaging by systematically inhibiting key inflammatory pathways.

## 3. Discussion

Alkannin is a key pharmacological component extracted from *Lithospermum erythrorhizon*, known for its anti-inflammatory and antioxidant properties. However, its specific protective mechanism against UVB-induced photoaging is not yet completely understood. This study preliminarily clarifies that Alk attenuates skin photoaging in both cellular and animal models by targeting the Keap1 protein and activating the Nrf2/HO-1 antioxidant signaling axis. Its mode of protection is pleiotropic, systematically intervening in the key pathological processes of photoaging by mitigating oxidative stress at the source, inhibiting inflammatory responses in the intermediate stage, and ultimately preventing extracellular matrix degradation. Serving as the body’s first line of defense, the skin is the first organ to encounter solar radiation. Among environmental factors, ultraviolet radiation (UVR) is the most notable contributor. The distinctive aging process it triggers is defined as “photoaging” [14]. UVR consists of UVA, UVB, and UVC, with UVB being the most impactful subtype in causing skin photoaging. Using a HaCaT cell-based photoaging model, this study aimed to uncover the mechanism behind Alk’s anti-photoaging action. The core mechanism of photoaging is known to involve UVR-induced excessive accumulation of intracellular ROS, which triggers oxidative stress, damages mitochondrial function, thereby promoting the release of senescence-inducing cytokines, such as matrix metalloproteinases, from keratinocytes and other cells [15,16]. These activated MMPs degrade collagen and elastin in the extracellular matrix [17], ultimately manifesting as a decline in skin elasticity and the emergence of wrinkles. Notably, key inflammatory cytokines like TNF-α amplify this signaling cascade. For instance, studies have shown that TNF-α-induced MMP-9 expression in macrophages depends on the upregulation of Cox-2 [18]. Our results demonstrate that Alk effectively reverses this series of UVB-induced cascade damages: it not only significantly enhances cell viability, reduces ROS accumulation, and improves overall cellular antioxidant capacity but also concurrently inhibits the UVB-induced production of TNF-α, Cox-2, and the downstream effector MMP-9, ultimately delaying the onset of cellular senescence phenotypes. These findings indicate that Alk’s protective effect involves multi-level intervention, mitigating oxidative stress at the source, suppressing the Cox-2/TNF-α/inflammatory response cascade midstream, and finally preventing extracellular matrix degradation. As mentioned above, Alk can combat photoaging and alleviate oxidative stress, but its specific mechanism remains unclear. The primary cause of photoaging is cellular oxidative stress damage induced by ROS. The transcription factor NRF2 can initiate endogenous antioxidant programs to counteract the oxidative damage caused by ROS. Numerous studies have shown that upregulation of NRF2 expression can mitigate oxidative stress and photoaging [19,20]. Although current research on Alk in skin remains limited, its isomer shikonin has demonstrated notable efficacy in treating cutaneous conditions such as burns and psoriasis. Previous studies have confirmed that shikonin modulates the Nrf2/HO-1 pathway, thereby performing its biological functions [12,21]. Our findings confirm that Alk interacts with Keap1 to suppress Nrf2 ubiquitination and degradation. As a consequence, Nrf2 accumulates and translocates into the nucleus, driving the subsequent upregulation of HO-1 expression. HO-1 production can directly neutralize ROS, thus mitigating oxidative damage at its source. A noteworthy finding is that even after knocking down Nrf2 using siRNA, Alk was still able to induce a partial upregulation of HO-1. This suggests that Alk may regulate HO-1 through Nrf2-independent parallel pathways. Literature reports indicate that its isomer shikonin can activate the HIF-1α pathway to influence HO-1 expression [22]. We speculate that Alk may possess multi-target characteristics, providing a new direction for exploring its complex pharmacological mechanisms. It should be noted that this speculation has not been experimentally validated in the current study. Future investigations are required to confirm this hypothesis through direct assessment of HIF-1α activation and the use of specific inhibitors. This study has certain limitations. First, it lacks comprehensive validation of gene expression changes in the Nrf2/HO-1 pathway at the transcriptional level (e.g., by qPCR). Second, the animal model is based on short-term, high-dose UVB irradiation, representing an acute photoaging model. This differs pathologically from natural photoaging models simulating long-term, low-dose UV exposure. Therefore, our findings primarily elucidate the intervention mechanism for acute UVB damage, and the long-term protective efficacy against chronic, cumulative photoaging requires further verification. Finally, physiological differences exist between the immortalized HaCaT cell line and primary keratinocytes. Future research should involve validation in primary cells and chronic photoaging models. Based on the preliminary results from the aforementioned cell models, this study further conducted experiments using an animal photoaging model to elucidate the anti-photoaging mechanism of compound Alk and systematically evaluate its potential application value. The main histological features of skin photoaging include epidermal thickening and collagen fiber degradation [23]. In vivo animal experiments demonstrated that Alk alleviated epidermal thickening and collagen fiber degradation in mice while enhancing the organism’s antioxidant capacity. In vitro data have already shown that compound Alk can inhibit inflammation and prevent extracellular matrix degradation. We further examined the abundance of related proteins through immunohistochemistry, and the results indicated that compound Alk reduced UV-induced dermal damage in mouse skin, specifically manifested as decreased protein levels of TNF-α, MMP-1, and MMP-9, along with preserved integrity of type III collagen. In the process of skin photoaging, TNF-α is widely recognized as a key driver, not only directly involved in photoaging but also capable of upregulating the expression of MMP-9 [24,25]. Notably, changes in the levels of MMP-1 and MMP-9 during this process are critical, as these two enzymes synergistically degrade the dermal extracellular matrix [26]: MMP-1 is primarily responsible for initiating the cleavage of native fibrillar collagen [27], while MMP-9 further degrades denatured collagen fragments and other basement membrane components [28], ultimately leading to the disruption of the dermal structural scaffold. Based on the above findings, we speculate that Alk may alleviate the degradation of type III collagen by blocking the TNF-α–MMPs cascade. As type III collagen is a key early component synthesized during tissue repair and regeneration, protecting it may help maintain dermal structure and promote a regenerative microenvironment [29]. It should be noted that this speculation has not yet been experimentally validated and awaits direct confirmation in future studies.

Transcriptome sequencing indicated that Alk treatment induced the downregulation of both TNF-α and IL-17 signaling pathways, with differentially expressed genes significantly enriched in the NF-κB pathway. Previous studies have confirmed that activation of the Nrf2 pathway can inhibit the activity of the NF-κB pathway [30], and NF-κB, in turn, activates inflammatory pathways including TNF-α [31,32]. Our experimental validation confirmed that Alk treatment effectively activated the intracellular Nrf2 signaling pathway and downregulated TNF-α expression. Integrating these findings, we propose that Alk may initially activate the intracellular Nrf2 antioxidant pathway, which in turn inhibits NF-κB activity, leading to an overall downregulation of downstream inflammatory signals, thereby ameliorating photoaging. However, direct experimental evidence regarding changes in NF-κB activity within this proposed signaling cascade still requires further investigation.

The present study has the following limitations. First, this study lacks comprehensive validation of gene expression changes in the Nrf2/HO-1 pathway at the transcriptional level (e.g., by qPCR). The animal model employed acute photoaging induced by short-term, high-dose UVB irradiation, which differs pathologically from chronic photoaging models that simulate long-term, low-dose UVB exposure. Therefore, our findings primarily elucidate the intervention mechanisms against acute UVB injury, while the long-term protective effects against chronic, cumulative photoaging require further validation. Additionally, the immortalized HaCaT cell line used in this study exhibits physiological differences from primary keratinocytes. Of particular note, compared to known Nrf2-activating polyphenols such as quercetin [33] and resveratrol [34], Alk exhibits higher cytotoxicity. This characteristic may be closely related to its naphthoquinone skeleton structure. Naphthoquinone compounds possess a highly electron-deficient quinone ring, which readily accepts a single electron to be reduced to a semiquinone radical. Subsequently, the semiquinone radical transfers an electron to molecular oxygen, generating superoxide anion and regenerating the quinone, thereby establishing a redox cycling process. This futile cycle continuously consumes reducing equivalents and generates reactive oxygen species (ROS), leading to the accumulation of hydrogen peroxide and oxidized glutathione, which in turn promotes the formation of cytotoxic mixed protein disulfides [35]. Furthermore, this study did not evaluate the effects of Alk on UVB-induced cyclobutane pyrimidine dimer (CPD) formation, DNA repair efficiency, or the apoptotic clearance of damaged cells. This is an important limitation of the present study. While Alk may protect cells from acute UVB-induced damage, it could also potentially enhance the survival of cells carrying DNA lesions (such as CPDs) that would otherwise undergo apoptosis. Delaying the removal of these damaged cells may allow mutations to accumulate, theoretically increasing the long-term risk of carcinogenesis. Therefore, the short-term photoprotective benefits of Alk should be carefully weighed against this potential safety concern. Meanwhile, the 6-day in vivo experimental period is too short to assess long-term efficacy or delayed toxicity, let alone to rule out cumulative oxidative damage or off-target effects that may result from long-term administration of naphthoquinone compounds. The absence of pharmacokinetic evaluation also leaves the absorption, distribution, metabolism, excretion, and bioavailability of Alk unclear. Future studies should address the above limitations from the following aspects: First, in light of the relatively higher cytotoxicity of Alk, its photoprotective effect at low concentrations can be exploited by employing nanoparticle encapsulation technology to achieve sustained release and targeted delivery, thereby reducing toxicity and improving safety. Second, chronic UVB exposure combined with long-term observation should be conducted to systematically evaluate the carcinogenic risk of Alk, along with pharmacokinetic studies to confirm the absence of significant absorption, distribution, and metabolic characteristics in vivo following topical application. Third, the photoprotective effects of Alk should be further validated in primary human keratinocytes and chronic photoaging animal models. Only after fully weighing the short-term photoprotective benefits against the long-term carcinogenic risks can the safety and feasibility of Alk for use in sunscreens or cosmetic products be comprehensively evaluated.

## 4. Materials and Methods

### 4.1. Reagents and Antibodies

Alkannin (SS8500, HPLC ≥ 98%) was obtained from Solarbio (Beijing, China). The UVB phototherapy lamp (SS-01B) was obtained from SIGMA (Shanghai, China). Carbomer 940, diethylene glycol monoethyl ether, and triethanolamine were sourced from Yuanye (Shanghai, China). The Lipo8000™ Transfection Reagent and Senescence β-Galactosidase Staining Kit were purchased from Beyotime Biotechnology (Shanghai, China). Hematoxylin, eosin staining solution, ROS Assay Kit, Co-Immunoprecipitation Kit, and ECL Chemiluminescent Substrate were obtained from Servicebio (Wuhan, China). The Masson’s Trichrome Stain Kit, Superoxide Dismutase Activity Assay Kit, and Malondialdehyde Content Assay Kit were acquired from Solarbio (Beijing, China). Primary antibodies against MMP-1 (GB15224-50) and MMP-9 (GB15132-50) were acquired from Servicebio (Wuhan, China). Antibodies against Nrf2 (16396-1-AP), COX-2 (66351-1-Ig), Keap1 (10503-2-AP), TNF-α (60291-1-Ig), HO-1 (10701-1-AP), β-actin (66009-1-Ig), and Lamin A/C (10298-1-AP) were obtained from Proteintech (Wuhan, China).

### 4.2. Cell Line and Culture Conditions

The immortalized human keratinocyte line HaCaT was obtained from Servicebio (Wuhan, China). Cells were cultured in DMEM containing 10% FBS and 1% penicillin/streptomycin at 37 °C with 5% CO_2_. At 80–90%, the cells were routinely subcultured using standard enzymatic digestion protocols.

### 4.3. Establishment of the Cellular Photoaging Model

To establish the optimal conditions for the HaCaT cell photoaging model, cells were covered with phosphate-buffered saline (PBS) and then exposed to UVB irradiation at doses of 240, 360, 480, and 600 mJ/cm^2^. UVB irradiation was performed using a UVB phototherapy lamp (SS-01B, SIGMA, Shanghai, China) with a peak wavelength of 311 nm (narrowband UVB). The lamp was turned on and preheated for 10 min to ensure stable output intensity. The UVB intensity was measured using a calibrated UV radiometer (LH126C, Lianhuicheng, China), and the distance from the UV source to the cell cultures was set to 10 cm, yielding an irradiation intensity of 1 mW/cm^2^. The irradiation dose (mJ/cm^2^) was calculated as intensity (mW/cm^2^) × irradiation time (s). After irradiation, the cells were cultured for an additional 24 h. To investigate the effect of Alk on HaCaT cell viability, cells were treated with Alk at concentrations ranging from 0 to 5 μM (0, 1, 2, 3, 4, 5 μM) for 24 h, and cell viability was subsequently assessed. To examine the protective effect of Alk against UVB-induced photoaging, HaCaT cells were pre-incubated with Alk at concentrations of 0, 0.25, 0.5, and 1 μM for 8 h. Following PBS coverage, cells were irradiated with UVB at a dose of 480 mJ/cm^2^ and then cultured for 24 h. Control cells were not exposed to UVB irradiation or Alk treatment. After each treatment, cell viability was determined using the Cell Counting Kit-8 (Solarbio, Beijing, China), which measures cell viability based on the reduction of WST-8 by cellular dehydrogenases to a water-soluble formazan dye.

### 4.4. Establishment of the Murine Skin Photoaging Model

The study was performed with the approval of Tarim University’s Animal Ethics Committee (No. PA20260108002). A total of 30 female BALB/c mice were obtained from the Xinjiang Medical University and subsequently housed in the designated animal quarters of Tarim University. A 1.2% blank hydrogel was prepared by dissolving carbomer 940 in distilled water and adjusting the pH to approximately 6 with triethanolamine. Alk was dissolved in diethylene glycol monoethyl ether to prepare a stock solution, which was then incorporated into the hydrogel base at specified concentrations. Following acclimatization, the mice were randomly allocated into six experimental cohorts of five animals each, using a random number table. Control (no treatment); UVB (UVB irradiation only); Vehicle (blank hydrogel + UVB); Alk-L (100 μM Alk hydrogel + UVB); Alk-M (200 μM Alk hydrogel + UVB); Alk-H (400 μM Alk hydrogel + UVB). The hydrogel (blank or Alk-formulated) was applied topically to the shaved dorsal skin 30 min prior to daily UVB irradiation. The irradiation intensity was 3.8 mW/cm^2^ for 10 min per day, conducted over 6 consecutive days. For downstream applications, mice were humanely sacrificed. Skin samples from the modeled dorsal area were then dissected, aliquoted, and stored accordingly.

### 4.5. Senescence-Associated β-Galactosidase and ROS Staining

Cellular senescence was evaluated with an SA-β-Gal staining kit. Intracellular ROS levels were measured using the DCFH-DA fluorescent probe method. Both assays were carried out in strict accordance with the manufacturer’s protocols. After staining, samples were observed and imaged under an inverted fluorescence microscope (Nikon, Tokyo, Japan).

### 4.6. Determination of Superoxide Dismutase (SOD) Activity and Malondialdehyde (MDA) Content

SOD activity and MDA content were determined by establishing detection assays according to their respective kit protocols. After the reactions were terminated, the liquid from each well was transferred into a 96-well plate, and absorbance was measured with a microplate reader following the set program.

### 4.7. Histological and Immunohistochemical Analysis

Skin tissues from the dorsal damaged area of mice were collected, rinsed with ice-cold PBS, and then trimmed to remove subcutaneous fat and connective tissue. We fixed the harvested tissues in 4% paraformaldehyde for 24 h. Subsequently, they were processed using an automated tissue processor for gradient dehydration, clearing, and paraffin infiltration, followed by embedding in paraffin blocks and sectioning. H&E and Masson’s trichrome staining were performed on tissue sections to evaluate epidermal thickness and collagen volume fraction, respectively. For immunohistochemistry, sections were incubated with primary antibodies against TNF-α (60291-1-Ig), Collagen Type III (22734-1-AP), MMP-1 (GB15224-50), or MMP-9 (GB15132-50) at 37 °C for 1 h. Then, the samples were washed three times with PBS.HRP-conjugated secondary antibodies (GB23303/GB23301) were applied, followed by visualization using a DAB substrate kit (G1212).

### 4.8. siRNA Transfection

According to the Lipo8000™ transfection reagent protocol, si-Nrf2 (Fenghui, Changsha, China) was mixed with the transfection reagent at the recommended ratio to form transfection complexes, which were then added to six-well plates pre-seeded with HaCaT cells. Owing to the low cytotoxicity of Lipo8000™, the transfected cells could be cultured continuously for 24 h without medium change.

### 4.9. Cellular Thermal Shift Assay (CETSA) and Isothermal Dose-Response Fingerprinting

HaCaT cells were plated in 10 cm dishes and incubated with either 1 μM Alk or a corresponding volume of DMSO as a vehicle control for 1.5 h. Subsequently, an adequate number of cells were harvested, resuspended in PBS supplemented with 1 mM PMSF, and distributed equally into 0.2 mL PCR tubes. The aliquoted suspensions were subjected to a 3-min heat challenge at graded temperatures (38, 41, 44, 47, 50, and 53 °C) in a PCR cycler. Post-heating, cells were lysed through three repeated freeze-thaw cycles in liquid nitrogen. Following centrifugation, the resulting supernatant was collected for subsequent Western blotting. For the isothermal dose–response experiment, a constant temperature of 47 °C was used, with Alk concentrations of 0.25, 0.5, and 1 μM, and DMSO as the control. The remaining experimental steps were consistent with the CETSA protocol.

### 4.10. Co-Immunoprecipitation (Co-IP)

Cells were treated with a combination of Alk (1 μM) and UVB irradiation for 1.5 h, while blank control cells remained untreated. Subsequently, cells were collected and lysed on ice using a lysis buffer supplemented with a protease inhibitor cocktail (YaMei, Shanghai, China). After centrifugation, the supernatant was carefully aspirated for further use. Following the Co-IP protocol, agarose beads were pre-coupled with either an anti-Nrf2 antibody or a control anti-IgG antibody. The prepared antibody-conjugated beads were then mixed with the protein samples and incubated overnight at 4 °C with gentle rotation. Beads were washed extensively, and bound proteins were eluted under acidic conditions for subsequent western blot analysis.

### 4.11. Western Blot Analysis

Cell lysis was performed with RIPA buffer (Solarbio, China) containing 1% PMSF to obtain total protein extracts. Protein concentration was assessed by the BCA assay, and denaturation was carried out at high temperature. Following SDS-PAGE separation, proteins were transferred electrophoretically to a PVDF membrane (Millipore, Burlington, MA, USA). After blocking for 15 min with a protein-free blocking reagent, the membrane was exposed to the corresponding primary antibody and incubated at 4 °C overnight. The primary antibodies used included: Nrf2 (16396-1-AP), COX-2 (66351-1-Ig), HO-1 (10701-1-AP), MMP-9 (GB15132-50), TNF-α (60291-1-Ig), Keap1 (10503-2-AP), with Lamin A/C (10298-1-AP) and β-actin (66009-1-Ig) serving as internal controls. After three washes with TBST buffer, the membranes were incubated for 1 h at room temperature with horseradish peroxidase-conjugated secondary antibodies obtained from Proteintech (SA00001-2) or Bioss (BC12124606; Woburn, MA, USA). Band visualization was performed, images were acquired using a gel imaging system, and the corresponding intensities were analyzed with the aid of ImageJ software (version 1.54f).

### 4.12. RNA Sequencing (RNA-Seq) Analysis

Skin tissue samples from the Control, UVB, and Alk-H groups were subjected to RNA-seq analysis to demonstrate the protective effect of Alk against skin photoaging. Three replicates of skin tissue were collected from each group. We entrusted Tsingke (Beijing, China) to perform library construction and sequencing on an Illumina platform. Data analysis involved aligning the acquired sequencing reads to the reference genome using the Hisat2 tool. Differential expression analysis was performed using DESeq2, with genes showing |log2FC| ≥ 1.0 and a Benjamini-Hochberg (BH) adjusted *p*-value < 0.05 considered significantly differentially expressed. Further analyses, including Venn diagrams, volcano plots, and KEGG pathway enrichment, were conducted based on these differentially expressed genes. For KEGG pathway enrichment analysis, significantly enriched pathways were identified using a BH-adjusted *p*-value < 0.05 as the threshold.

### 4.13. Statistical Analysis

All data are presented as mean ± standard deviation (SD). Statistical analysis was performed using GraphPad Prism software (version 9.0, USA). Comparisons between two groups were analyzed using the unpaired Student’s *t*-test. Comparisons among multiple groups were performed using one-way analysis of variance (ANOVA). A *p*-value of less than 0.05 was considered statistically significant.

## 5. Conclusions

This study elucidates that Alk effectively alleviates UVB-induced skin photoaging by targeting Keap1 to activate the Nrf2/HO-1 pathway. To the best of our knowledge, direct targeting of the Keap1 protein by Alk has not been previously reported. This protective effect is primarily mediated through the following mechanisms: attenuating oxidative stress, suppressing inflammatory reactions, and delaying extracellular matrix degradation. This study establishes a solid theoretical foundation for the development of Alk as a potential botanical-derived active ingredient to prevent skin photoaging.

## Data Availability

The original contributions presented in this study are included in the article. Further inquiries can be directed to the corresponding author.

## Abbreviations

The following abbreviations are used in this manuscript:

UVB	Ultraviolet B
HO-1	Heme oxygenase-1
ROS	Reactive Oxygen Species
TNF-α	Tumor necrosis factor-alpha
Nrf2	Nuclear factor erythroid 2-related factor 2
Keap1	Kelch-like ECH-associated protein 1
